# White matter hypointensities and hyperintensities have equivalent correlations with age and CSF β‐amyloid in the nondemented elderly

**DOI:** 10.1002/brb3.1457

**Published:** 2019-11-06

**Authors:** Ke Wei, Thao Tran, Karen Chu, Matthew T. Borzage, Meredith N. Braskie, Michael G. Harrington, Kevin S. King

**Affiliations:** ^1^ Advanced Imaging and Spectroscopy Center Huntington Medical Research Institutes Pasadena CA USA; ^2^ Neuroscience Department Huntington Medical Research Institutes Pasadena CA USA; ^3^ Fetal and Neonatal Institute Division of Neonatology Children's Hospital Los Angeles Department of Pediatrics Keck School of Medicine University of Southern California Los Angeles CA USA; ^4^ Department of Neurology Imaging Genetics Center Stevens Neuroimaging and Informatics Institute Keck School of Medicine University of Southern California Los Angeles CA USA

**Keywords:** aging, Alzheimer's disease, hyperintensity, hypointensity, leukoaraiosis, white matter lesion

## Abstract

**Introduction:**

T1‐ and T2‐weighted sequences from MRI often provide useful complementary information about tissue properties. Leukoaraiosis results in signal abnormalities on T1‐weighted images, which are automatically quantified by FreeSurfer, but this marker is poorly characterized and is rarely used. We evaluated associations between white matter hyperintensity (WM‐hyper) volume from FLAIR and white matter hypointensity (WM‐hypo) volume from T1‐weighted images and compared their associations with age and cerebrospinal fluid (CSF) β‐amyloid and tau.

**Methods:**

A total of 56 nondemented participants (68–94 years) were recruited and gave informed consent. All participants went through MR imaging on a GE 1.5T scanner and of these 47 underwent lumbar puncture for CSF analysis. WM‐hypo was calculated using FreeSurfer analysis of T1 FSPGR 3D, and WM‐hyper was calculated with the Lesion Segmentation Toolbox in the SPM software package using T2‐FLAIR.

**Results:**

WM‐hyper and WM‐hypo were strongly correlated (*r* = .81; parameter estimate (p.e.): 1.53 ± 0.15; *p* < .0001). Age was significantly associated with both WM‐hyper (*r* = .31, p.e. 0.078 ± 0.030, *p* = .013) and WM‐hypo (*r* = .42, p.e. 0.055 ± 0.015, *p* < .001). CSF β‐amyloid levels were predicted by WM‐hyper (*r* = .33, p.e. −0.11 ± 0.044, *p* = .013) and WM‐hypo (*r* = .42, p.e. −0.24 ± 0.073, *p* = .002). CSF tau levels were not correlated with either WM‐hyper (*p* = .9) or WM‐hypo (*p* = .99).

**Conclusions:**

Strong correlations between WM‐hyper and WM‐hypo, and similar associations with age, abnormal β‐amyloid, and tau suggest a general equivalence between these two imaging markers. Our work supports the equivalence of white matter hypointensity volumes derived from FreeSurfer for evaluating leukoaraiosis. This may have particular utility when T2‐FLAIR is low in quality or absent, enabling analysis of older imaging data sets.

## Graphical abstract

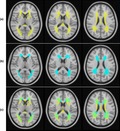

## 1 INTRODUCTION

**Figure 1:**
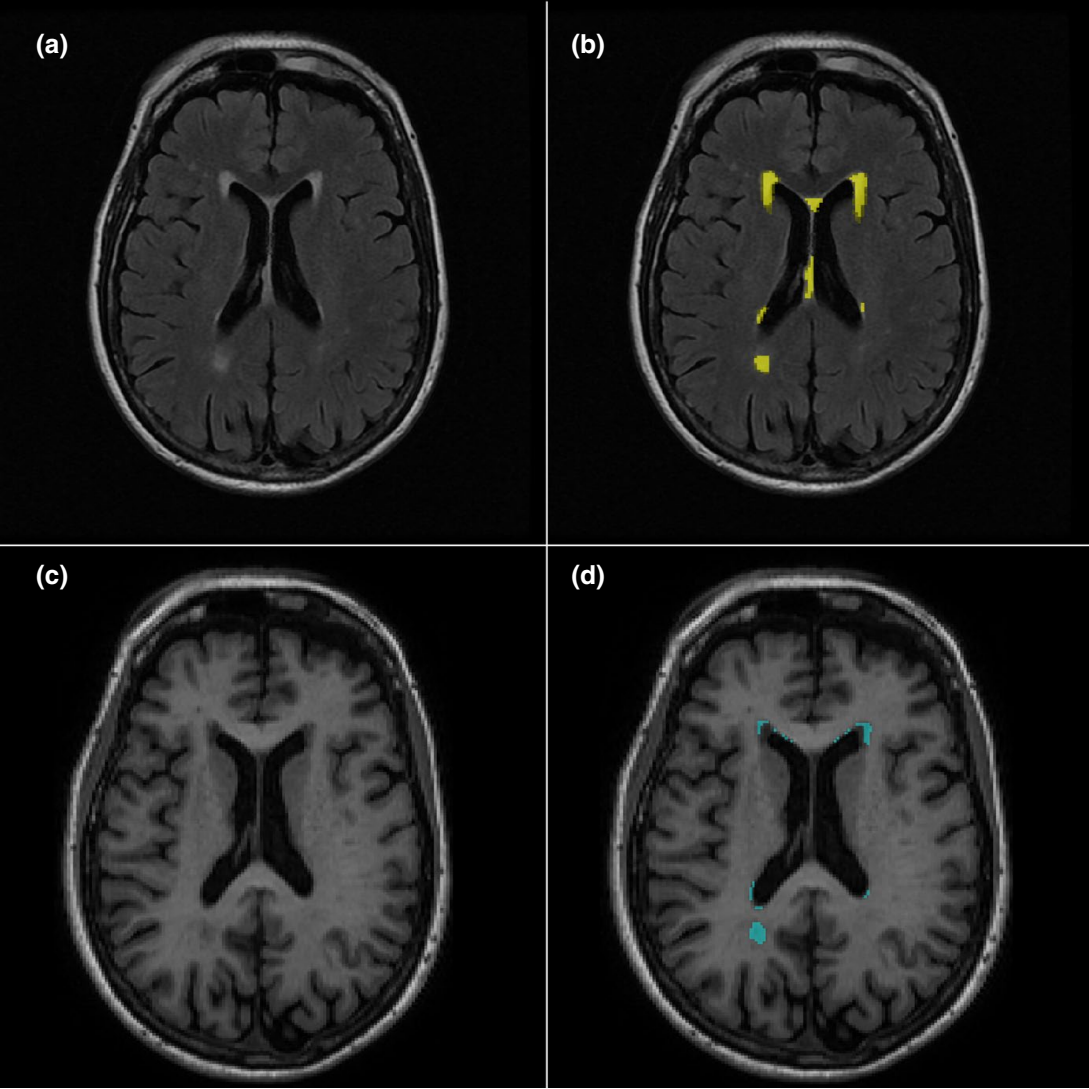
White matter lesions appear bright (WM‐hyper) on T2‐FLAIR (a) and dark (WM‐hypo) on T1‐FSPGR (c) from a representative individual. Automatically identified WM lesions are shown overlaid on anatomic images with a yellow mask for WM‐hyper in (b) and with a blue mask for WM‐hypo in (d)

Chronic vascular insult is an important biomarker for cognitive impairment (Gorelick et al., 2011) where damage accumulates silently for decades before the onset of clinically identifiable dementia symptoms (Brown & Thore, 2011). Early detection of microvascular disease is therefore critical to identify and guide efforts to prevent the onset of dementia. Neuroimaging is an essential tool in the evaluation of age‐related accumulation of small vessel disease that contributes to vascular insult in the brain, which shows up as white matter lesions. The term “leukoaraiosis” was used to describe such phenomenon by Hachinski et al. to these white matter lesions as a descriptive placeholder to prevent “premature” assumption of pathology and to “encourage the search for causes” (Hachinski, Potter, & Merskey, 1986); the Greek root leuko‐ means white and ‐araiosis is a rarefication, meaning a reduced density. On computed tomography (CT) and T1‐weighted magnetic resonance imaging (MRI), white matter lesions appear dark and have been termed white matter hypointensities (WM‐hypo), while on fluid‐sensitive MRI sequences such as fluid‐attenuated inversion recovery (FLAIR), they appear bright and are referred to as white matter hyperintensities (WM‐hyper) as shown in Figure 1. Most research has utilized FLAIR in particular because of the increased conspicuity of WM lesions after the suppression of signal from the cerebrospinal fluid. Previously, the presence of abnormalities on spin‐echo T1‐weighted sequences which had lower sensitivity for lesion detection was used as a marker of more severe insult (Sinnecker et al., 2012). Continued sequence development in MRI led to the advent of ultrafast gradient‐echo sequences, such as T1 magnetization prepared rapid acquisition gradient echo (MPRAGE), which increased conspicuity of WM‐hypo on T1 (Mistry et al., 2011). Currently, there is limited understanding of similarities or differences between WM‐hypo derived from modern 3D gradient‐echo sequences and WM‐hyper derived from T2‐FLAIR (Dadar et al., 2018).

Assessment of T1 and T2 values may provide additional specificity about the underlying pathology and severity of white matter lesions. For example, during development, the myelinating white matter undergoes temporally distinct phases of signal intensity changes on T1‐ and T2‐weighted images (Ashikaga, Araki, Ono, Nishimura, & Ishida, 1999), reflecting the complex microstructural origin of MRI signals. Early in development, myelination results in an increased signal on T1 approaching the adult state, while the signal on FLAIR remains bright, reflecting persistent elevations in free water (Holland, Haas, Norman, Brant‐Zawadzki, & Newton, 1986). Changes on T1 and FLAIR with leukoaraiosis may also represent distinct, though related, changes in the white matter as is seen during development. Thus, WM‐hyper and WM‐hypo volumes might be closely despite actually measuring something different (Erkinjuntti et al., 1987). Though age‐related WM lesions are commonly attributed to chronic ischemic cerebrovascular disease, they are nonspecific. A growing number of studies (King, 2014; Lee et al., 2016; Pietroboni et al., 2018) are finding direct correlations of WM disease with β‐amyloid and tau accumulation, supporting significant overlap between WM lesion development and neurodegeneration (Erten‐Lyons et al., 2013; McAleese et al., 2015). Ischemic white matter lesions may indicate a vascular susceptibility to AD (Hughes et al., 2013; King, 2014; King et al., 2014). Newer evidence points to other pathologic pathways whereby AD itself causes damage to the white matter tracts or results in secondary axonal degeneration due to gray matter damage (Amlien & Fjell, 2014).

Automated tools may provide significant benefits in generating reproducible and accurate assessments of white matter disease burden. Methods for automated assessment of FLAIR WM‐hyper (Hulsey et al., 2012) have been developed for research, but no single approach has achieved widespread adoption. Recently, FDA‐approved programs like LesionQuant began offering an automatic evaluation of WM‐hyper. High contrast and high‐resolution T1‐weighted images are the primary input requirement for automated brain segmentation programs such as FreeSurfer (Bruce Fischl, 2012) and the related FDA‐approved NeuroQuant (Cortech Labs, San Diego). Both programs report WM‐hypo volumes, but information of WM‐hypo is less frequently used than WM‐hyper. A motivation for our study was to evaluate whether WM‐hypo easily obtained from the widely utilized FreeSurfer program from T1 images available in most imaging data sets would be equivalent to WM‐hyper. Since FreeSurfer is already widely used in research, this has value as a standardized measure for better comparison between studies. WM‐hypo assessment may also be advantageous in analyzing white matter disease in older data sets, such as those from the Alzheimer's Disease Neuroimaging Initiative's initial cohort (ADNI 1) that may have high‐resolution T1 but may not have FLAIR.

In this study, we sought to assess whether WM‐hypo and WM‐hyper are equivalent markers of WM damage in normal aging and early neurodegenerative disease by evaluating their associations with age and cerebrospinal fluid (CSF) levels of β‐amyloid and tau. We hypothesized that WM‐hypo and WM‐hyper volumes would be highly correlated, but that volumes of WM‐hyper would be more extensive over a larger region when compared with WM‐hypo. We also hypothesized that WM‐hypo and WM‐hyper would be increased with age and would reflect neurodegeneration as revealed by abnormal CSF β‐amyloid and tau levels.

## 2 METHODS

### 2.1 Sample population

In this IRB‐approved study with written informed consent, 56 nondemented participants were examined. All participants were recruited through newspaper article, local senior centers visit, and word of mouth. All participants underwent MR imaging and cognitive testing but only 47 consented to lumbar puncture for CSF collection (Harrington et al., 2013).

### 2.2 Cognitive assessment

Study participants were included if they were classified as cognitively healthy (CH) or had mild cognitive impairment (MCI) after medical and neuropsychological assessment using the Uniform Data Set‐3 criteria (Weintraub et al., 2018) of the National Alzheimer's Coordinating Center after consensus clinical conference as previously described for this study cohort (Harrington et al., 2013). The CH group was asymptomatic with Clinical Dementia Rating (CDR) of zero and neuropsychological measurements within one standard deviation of mean according to published normative values. Mild cognitive impairment (MCI) participants were those individuals with CDR of 0.5 and fulfilled the current MCI criteria (Mayeux et al., 2011; Seshadri et al., 2011).

### 2.3 MRI data acquisition

All brain imaging was performed at the Advanced Imaging and Spectroscopy Center of the Huntington Medical Research Institutes (Pasadena, CA) on a 1.5 Tesla General Electric (GE) clinical scanner using an 8‐channel high‐resolution head coil. Standard imaging consisted of T1‐weighted 3D fast‐spoiled gradient echo (FSPGR) (echo time (TE) 3.9 ms; repetition time (TR) 8.9 ms; inversion time (TI) 500 ms; slice thickness (ST) 1.2 mm; matrix 192 × 192; NEX 1) and T2‐weighted fluid‐attenuated inversion recovery (FLAIR) (TE 124 ms; TR 9,500 ms; TE 2,250 ms; ST 4 mm; matrix 310 × 224; NEX 1). All images were reviewed by a board‐certified neuroradiologist to rule out masses and lesions.

### 2.4 White matter hyperintensity analysis

WM‐hyper lesion load was quantified using the Lesion Growth Algorithm (LGA) in the Lesion Segmentation Toolbox 2.0.1.5 (LST, http://www.statistical-modelling.de/lst.html) with suggested threshold of 0.3 (Schmidt et al., 2012). Lesion probability maps and calculated WM lesion load (μl) were produced.

### 2.5 White matter hypointensity analysis

WM‐hypo lesion load (μl) using FreeSurfer image analysis suite 6.0 (http://surfer.nmr.mgh.harvard.edu/) using T1‐weighed images. WM‐hypo is identified by using spatial intensity gradients across tissue classes (Desikan et al., 2006; B. Fischl et al., 2004).

### 2.6 WM lesion load on Standard Space

WM‐hyper and WM‐hypo masks of these 56 participants were transposed onto the standard space using SPM12 with a voxel size of 1 mm. The WM‐hyper and WM‐hypo masks in standard space were added together to create a group distribution mask showing all WM‐hyper lesions in the cohort. The group mask intensity thresholds were set to greater than 1 to limit noise.

### 2.7 CSF data acquisition

After an overnight fast, lumbar CSF was obtained between 8:00 a.m. and 10:00 a.m. and immediately examined for cells and total protein. CSF was stored in 1 ml aliquots at −80°C and then thawed for β‐amyloid (Aβ_42_) and total tau group assays (Harrington et al., 2013) using a sandwich enzyme‐linked immunosorbent assay kit (Innotest β‐amyloid (1–42) and Innotest hTAU‐Ag, Innogenetics, Gent, Belgium) according to the manufacturer's protocol.

### 2.8 Statistical analysis

Statistical analyses were performed using JMP, V13.0 (SAS Institute, Inc.). The distribution of WM‐hyper and WM‐hypo volumes was described by range and quantile. Statistical significance was set at *p *< .05 using two‐tailed tests with multiple comparison correction using the Benjamini‐Hochberg False Discovery Rate (FDR) (Benjamini & Hochberg, 1995), where *p*‐values presented are already FDR corrected. Due to the skewed distribution of WM‐hyper and WM‐hypo volume, log transformation of WM‐hyper, WM‐hypo, β‐amyloid, and tau is applied for parametric analysis. General linear regression was used to evaluate the relationship between WM‐hyper and WM‐hypo. Age is a robust universal predictor of WM lesions, so we then evaluated the association of age as independent predictor variables to WM‐hyper and WM‐hypo as dependent variables, and in a second model, we further adjusted for cognitive status. We then evaluated WM lesions as predictor variables for their associations with CSF markers (β‐amyloid and tau) as dependent outcome variables and if significant we evaluated an additional model adjusted for age and cognitive status.

## 3 RESULTS

Demographic information is shown in Table 1. Among 56 participants with a brain MRI, there were 39 CH and 17 MCI. In the subset of 47 with lumbar puncture for CSF analysis, there were 34 CH and 13 MCI. The ratio for CH and MCI was not significantly different between those with MRI only and those with MRI and CSF (chi‐square *p *= .8).

**Table 1:** Study population demographics and clinical parameters

Parameters		
Sample size		56
Cognitive status	CH: MCI	39:17
Sex	F:M	40:16
Age	Mean	80.7
	Media	80
	IQR	75.5–86
WM‐Hyper (µl)	Mean	7,197
	Media	4,173
	IQR	1,856–8,438
WM‐Hypo (µl)	Mean	6,407
	Media	3,728
	IQR	2,424–7,736
Beta‐Amyloid^a^ (pg/ml)	Mean	761
	Media	742.
	IQR	553–873
Tau^a^ (pg/ml)	Mean	309
	Media	287
	IQR	172–383

a Sample size is 47 for CSF markers (34CH, 13 MCI).

### 3.1 Distributions of WM‐hyper and WM‐hypo

**Figure 2:**
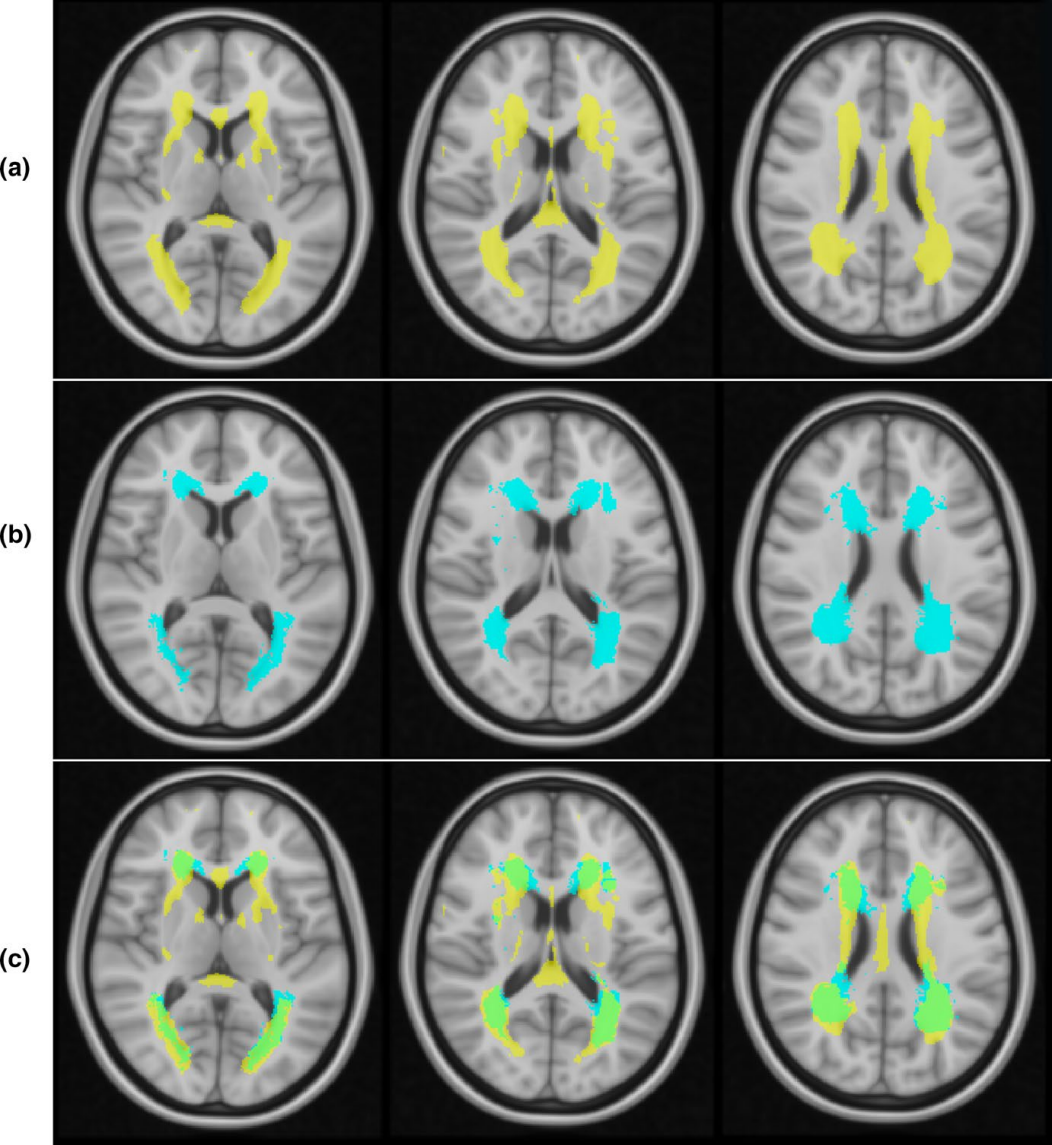
The anatomic locations of WM lesions are shown projected onto standard space on 3 representative axial images as follows: (a) WM‐hyper in yellow, (b) WM‐hypo in blue, and (c) superimposed WM‐hyper and WM‐hypo with overlap in green

WM‐hyper ranges from 18.28 to 43,862.3 μl with interquartile range of 1,855.70–8,438.03 μl and median of 4,173.40 μl. WM‐hypo ranges from 1,098.80 to 33,761.70 μl with interquartile range of 2,423.5 to 7,735.93 μl and median of 3,728.15 μl. WM‐hyper and WM‐hypo spatial distribution are shown in Figure 2. WM‐hyper occurs at locations of WM‐hypo but also involves additional brain regions, such as the fornix and internal capsule.

### 3.2 WH‐hyper verses WM‐hypo volumes

**Figure 3:**
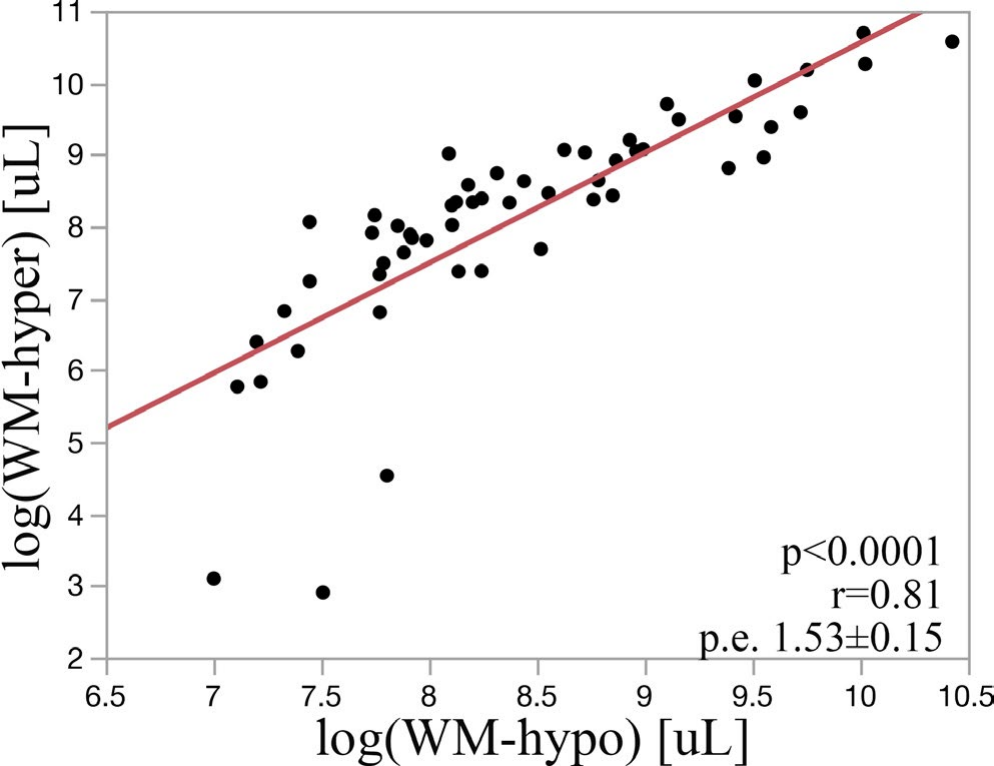
WM‐hyper and WM‐hypo are strongly linearly correlated

Log transformed WM‐hypo was strongly associated with WM‐hyper (*r* = .81; parameter estimate (p.e.): 1.53 ± 0.15; *p* < .0001) as shown in Figure 3. Across the population, WM‐hyper volumes tended to be greater and distributed over a larger region than WM‐hypo as shown in Figure 2a,b. This observation is more obvious when overlapping WM‐hyper and WM‐hypo distributions together (Figure 2c). Conversely, WM‐hypo tended to involve a smaller region that was entirely within the area occupied by WM‐hyper.

### 3.3 Age‐related differences in WM‐hyper and WM‐hypo

**Figure 4:**
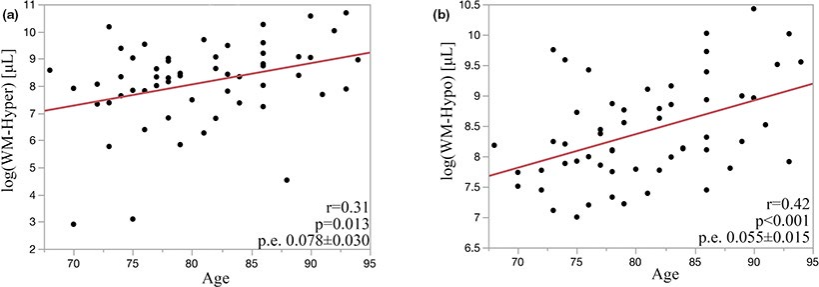
Increase in WM‐hyper (a) and WM‐hypo (b) shows a significant correlation with age

Figure 4 shows WM lesion volume plotted as a function of age. Age had a significant correlation with WM‐hyper (Figure 4a; *r* = .31, p.e. 0.078 ± 0.030, *p* = .013) and with WM‐hypo (Figure 4b; *r* = .42, p.e. 0.055 ± 0.015, *p* < .001). Adjusting for cognitive status did not significantly alter age association with WM‐hyper (0.080 ± 0.03, *p* = .01) or WM‐hypo (0.056 ± 0.016, *p* < .001).

### 3.4 WM‐hyper and WM‐hypo association with CSF β‐amyloid and tau

**Figure 5:**
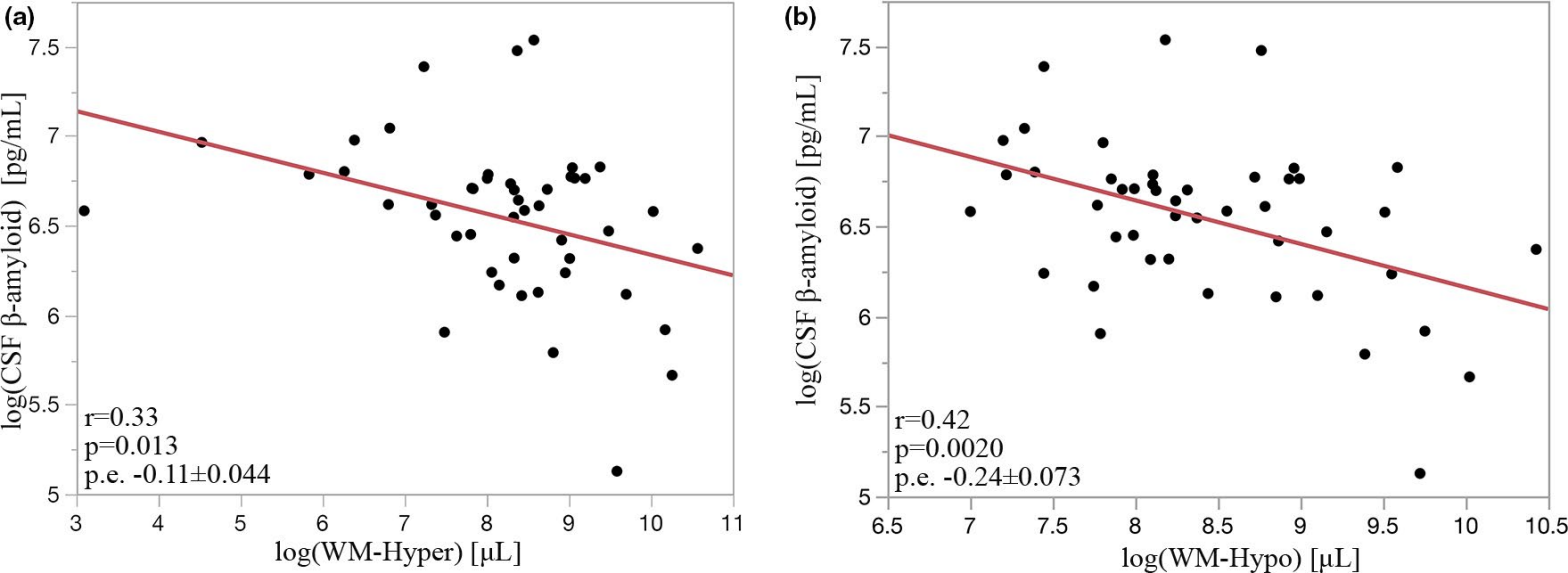
Increased WM‐hyper (a) and WM‐hypo (b) were strongly correlated with low levels of CSF β‐amyloid, supporting a link between white matter damage and changes associated with early Alzheimer's disease pathology

**Figure 6:**
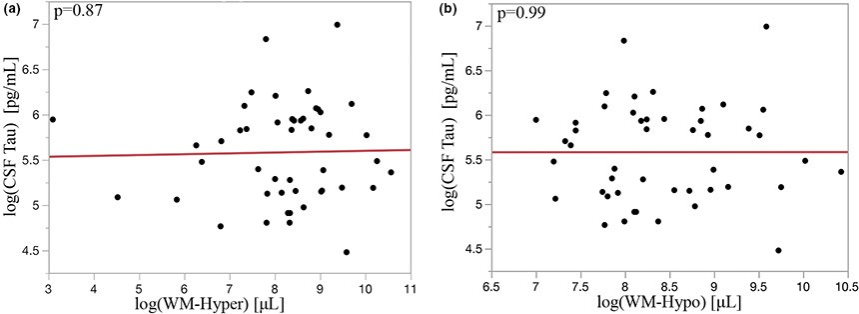
WM‐hyper (a) and WM‐hypo (b) showed a trend toward higher values among those with greater CSF Tau concentrations

Correlations of WM‐hyper and WM‐hypo with CSF markers were evaluated and if significant were further evaluated in a linear model adjusted for age and cognitive status. Relationships of WM‐hyper and WM‐hypo with β‐amyloid are shown in Figure 5. β‐Amyloid accumulation in the brain coincides with a decrease in CSF amyloid. Lower CSF β‐amyloid concentrations, the more pathological condition, were predicted by greater WM‐hyper (Figure 5a; *r* = .33, p.e. −0.11 ± 0.044, *p* = .013) and WM‐hypo (Figure 5b; *r* = .40, p.e. −0.24 ± 0.073, *p* = .002) volumes. Adjusting for age and cognitive status did not significantly alter prediction of β‐amyloid by WM‐hyper (−0.12 ± 0.05, *p* = .016) and WM‐hypo (−0.26 ± 0.08, *p* = .003). There was no correlation of CSF tau with WM‐hyper (Figure 6a; *p* = .9) or WM‐hypo (Figure 6b; *p* = .99).

## 4 DISCUSSION

Our study results suggest WM‐hypo and WM‐hyper have equivalent correlations when used as neuroimaging markers of leukoaraiosis, with a high degree of correlation between them and similar associations with age and CSF β‐amyloid and tau. WM‐hypo lesion volumes quantified from T1 gradient‐echo sequences using FreeSurfer program are strongly correlated with WM‐hyper volumes derived from T2‐FLAIR. However, we observed some differences in spatial distribution: WM‐hypo distribution is predominantly in the periventricular region, whereas WM‐hyper extended more into the deep white matter (Figure 2). The distribution we observed in our study is very similar to that reported by Riphagen et al. (2018), supporting generalizability. WM‐hyper and WM‐hypo were generally equivalent in predicting the presence of abnormal CSF β‐amyloid, however, neither showed any association with CSF tau. Overall, our findings support that WM‐hypo derived from T1‐weighted images obtained for 3D volumetric analysis are generally equivalent to WM‐hyper derived from T2‐FLAIR.

Log transformed WH‐hyper and WM‐hypo volumes were highly correlated, with an adjusted *r* = .81, as shown in Figure 3. The largest deviations from the fit line between WM‐hypo and WM‐hyper were seen among the lower range values of WM‐hyper lesion. These discrepancies among smaller WM lesion volumes are not likely to be clinically significant as impairment is mostly associated with large lesion volumes (King, Peshock, et al., 2013). As WM lesion volumes increase, we observe a better fit between WM‐hypo and WM‐hyper as shown by less deviation of points from the regression line. We also observe that WM‐hyper increase in volume is greater than the corresponding value of WM‐hypo. This supports our assertion that for a given lesion volume, WM‐hypo corresponds to more advanced disease as compared to WM‐hyper. This further corroborates with the spatial distribution of WM‐hyper and WM‐hypo as shown in Figure 2. The area of WM‐hypo involvement is almost entirely within the region where WM‐hyper occurs in the periventricular white matter. WM‐hyper has additional involvement within the deep white matter, which may correspond to an earlier stage of disease development (Schmahmann, Smith, Eichler, & Filley, 2008). In future longitudinal studies, we will assess whether WM‐hypo later spread to involve areas that currently only demonstrate WM‐hyper. Though WM‐hyper is nonspecific and associated with many factors, age is consistently the strongest predictor of lesion accumulation in community‐based studies (King, Chen, et al., 2013; Zhong & Lou, 2016). We found the expected linear correlation between larger WM‐hyper and WM‐hypo (Figure 4) volume with greater age. It suggests that evaluation of T1 has utility in demonstrating the presence of age‐related white matter changes in the absence of a T2‐FLAIR sequence.

Both WM‐hypo and WM‐hyper were shown to be increased among those with lower CSF β‐amyloid in our cohort of nondemented individuals (Figure 5). The presence of this association has been interpreted by some as suggestive that damage to small blood vessels within the brain may promote risk for Alzheimer's disease (Iadecola, 2010). Recent work has suggested that amyloid accumulation associated with AD neurodegeneration may also contribute to the formation of WM lesions independent of vascular diseases (Scott et al., 2016). WM lesions are present in autosomal dominant familial Alzheimer's disease (Lee et al., 2016) even at younger ages when microvascular disease is not generally prevalent (King et al., 2014). AD‐related damage may cause white matter damage through Wallerian degeneration occurring secondary to neuronal insult (McAleese et al., 2017). A recent study by Pietroboni et al. (2018) found abnormal β‐amyloid to be the best predictor of WM lesion load within a cohort with AD. Low CSF β‐amyloid levels indicate impaired clearance from, and accumulation within, the brain and may detect abnormalities earlier than positron emission tomography (PET) (Palmqvist, Mattsson, & Hansson, 2016). An important difference in our current study is that we excluded individuals with dementia, demonstrating association of WM lesion with earlier stages of AD pathology in a cohort consisting mostly of cognitively intact individuals. Consistent results produced by comparing WM‐hypo and WM‐hyper directly denote them to be comparable markers of leukoaraiosis and overall suggest tight pairing between distinct changes occurring in the white matter driving T1‐ and T2‐weighted signal changes (Simpson et al., 2007).

Despite showing statistically significant associations with β‐amyloid, we observed no associations with tau for either WM‐hyper or WM‐hypo (Figure 6), but work by McAleese et al. (2015) found a closer independent correlation between WM lesion load in AD with tau than β‐amyloid. This difference may be related to a lower prevalence of tau in our cohort, which includes primarily cognitively intact, nondemented individuals (Harrington et al., 2013). CSF tau increases as cognitive health declines and tends to be a more accurate marker of later AD stage (Shim & Morris, 2011). It is therefore possible that tau may become more closely correlated with WM lesion volume as our cohort ages.

A recent study by Dadar et al. (2018) provided the initial proof of T1‐weighted assessment of WM‐hypo lesions in comparison with hyperintense lesions identified on T2‐weighted, proton density (PD), or FLAIR. We build upon this work here in several respects. First, the prior study used an in‐house Random Forest classifier detection program for WM‐hypo and manual segmentation for WM‐hyper, and it is unclear how these findings will generalize to other methods. Here, we use freely available automated segmentation tools for the identification of both WM‐hypo and WM‐hyper. Second, in our current work, we provide more information about the correlation of white matter lesions with the presence of neurodegeneration as indicated by CSF levels of β‐amyloid and tau.

### 4.1 Limitations

There are several limitations to this study. First, we only have a cohort of 56 nondemented subjects. The small sample size may not fully represent the general population; however, the result is the first step toward analyzing a larger data set. Second, while we compared WM‐hypo and WM‐hyper, differences in the underlying algorithms for detecting lesions may also underlie differences we observed rather than actual differences in tissue pathology. We used the Lesion Segmentation Tool, an automated method for quantifying FLAIR‐hyperintense WM lesions which first relies on tissue segmentation performed by SPM using the intensity values in T1‐weighted anatomic images to identify tissues as gray matter, white matter, and CSF. WM‐hypo is generated by FreeSurfer which uses a more sophisticated algorithm guided by an anatomic atlas for identifying gray matter, WM, and CSF. Third, we utilize cross‐sectional comparisons of WM‐hyper and WM‐hypo with other markers and cannot assess causation. Finally, in this initial work, we compare only global WM‐hyper and WM‐hypo volumes. Future studies are needed to take into account spatial distribution and determine if overlapping or nonoverlapping WM‐hyper and WM‐hypo lesions may provide additional information about lesion severity or specific associations with outcomes.

## 5 CONCLUSION

Automated assessment of WM‐hypo volume is closely associated with WM‐hyper and both provide equivalent measures of leukoaraiosis progression with aging and prediction of abnormal CSF β‐amyloid. WM‐hypo volumes generated by FreeSurfer, which are currently rarely employed, can serve as a meaningful measure of white matter insult. This may facilitate retrospective analysis of older imaging data sets or serve as a standardized technique for comparison of WM lesion severity in existing studies using a widely available automated technique with a corresponding FDA‐approved version available for clinical use.

## CONFLICT OF INTEREST

There are no conflicts of interest to disclose.

## Data Availability

The data that support the findings of this study are available from the corresponding author upon reasonable request. Data collected at Huntington Medical Research Institutes is a contributor to ADNI's data collection.
